# Effects of L-Proline on the Stability of Mulberry Anthocyanins and the Mechanism of Interaction between L-Proline and Cyanidin-3-*O*-Glycoside

**DOI:** 10.3390/molecules29194544

**Published:** 2024-09-25

**Authors:** Haipeng Cui, Xianbao Li, Yuan Ji, Shengxu Zhao, Jianting Yang

**Affiliations:** 1 School of Food Engineering, Anhui Science and Technology University, Chuzhou 233100, China; chp@ahstu.edu.cn (H.C.); yjs2023253@ahstu.edu.cn (Y.J.); yjs2023294@ahstu.edu.cn (S.Z.); 2 School of Food and Pharmacy, Shanghai Zhongqiao Vocational and Technical University, Shanghai 201514, China; 19856981229@163.com

**Keywords:** mulberry anthocyanins, L-proline, C3G, molecular docking, molecular dynamics modes

## Abstract

The protective effects of L-aspartic acid, L-valine, and L-proline on the stability of mulberry anthocyanins were investigated. Results showed that L-aspartic acid, L-valine, and L-proline significantly enhanced (*p* < 0.05) the stability of mulberry anthocyanins under constant light or ascorbic acid (AA). L-Proline had the best protective effect against anthocyanin degradation. The interaction between L-proline and cyanidin-3-*O*-glucoside (C3G) through hydrogen bonding and van der Waals forces, which improved the stability of C3G, was confirmed using FT-IR, ^1^H NMR, XRD, and molecular docking analyses, as well as molecular dynamics modes. In vitro digestion experiments yielded that both 2,2-Diphenyl-1-picrylhydrazyl (DPPH) and 2,2′-Azino-bis(3-ethylbenzothiazoline-6-sulfonic acid) (ABTS) free radical scavenging capacities of the C3G/Pro group were increased in the intestinal fluid (*p* < 0.05). The above findings suggest that L-proline effectively slowed down the degradation of mulberry anthocyanins, and that it could be used as an auxiliary pigment and food additive to extend the optimal flavor period of products containing mulberry anthocyanins, and can improve the bioavailability of mulberry anthocyanins.

## 1. Introduction

Mulberry is the polyfloral fruit of the mulberry tree, a perennial woody plant in the Moraceae family that is widely distributed in China [1]. Mulberry is rich in dietary fiber, sugar, anthocyanins, proteins, and a variety of essential amino acids, and it is the first batch of varieties selected by the Ministry of Health of China’s “Medicine and Food” list [2]. Cyanidin-3-*O*-glucoside (C3G), one of the main bioactive ingredients in mulberry, has a variety of physiological active functions such as hypoglycemic, antioxidant, and anti-lipogenetic activities [3]. C3G is a water-soluble pigment that exhibits high solubility in water. However, its stability is relatively poor when existing alone, as the phenolic hydroxyl groups in its structure are prone to oxidation, leading to changes in its properties [4]. Notably, it possesses strong antioxidant activity, capable of eliminating free radicals within the body and protecting cells from oxidative damage [5]. The molecular structure of anthocyanins is prone to breakdown under the influence of conditions such as sunlight, heat, and ascorbic acid (AA), resulting in discoloration or reduced biological activity, which limits the application of anthocyanins [6]. The juice and jam product industry occupies the majority of the mulberry processing market. The low degree of processing involved in mulberry production (e.g., fruit juice, jam, fruit wine, etc.) results in a relatively low production value for this commodity. However, the intensive processing of mulberry (e.g., ultrafine powder, freeze-drying, extracts, etc.) affects the bioavailability and stability of mulberry anthocyanins. Therefore, it is necessary to improve the stability of mulberry anthocyanins and promote the intensive processing of mulberry to increase the added value of mulberry.

Multiple studies have shown that amino acids can be used as co-pigmenting substances added to anthocyanin-containing foods to improve the shelf-life and stability of anthocyanins. The study indicated that L-aspartic acid, L-proline, and L-valine effectively slowed anthocyanin degradation and enhanced the stability of total anthocyanins and C3G under high-temperature conditions [7,8]. It has been shown that van der Waals forces and intermolecular hydrogen bonds formed between anthocyanins and amino acids, thereby inhibiting the nucleophilic attack of water molecules on anthocyanins and thus increasing anthocyanin stability, prolonging their freshness, and maintaining their color and nutritional value [9,10]. Therefore, the addition of amino acids may contribute to the interaction between anthocyanins and amino acids and improve the stability of mulberry anthocyanins. L-Proline not only contributes to muscle and joint health and skin health, but it is an important component of collagen synthesis, playing an important role in the body’s protein synthesis [11]. However, few studies have examined the effect of L-proline on the stability of mulberry anthocyanins under constant light or in the presence of ascorbic acid (AA).

During the preparation and storage of mulberry products, they are usually exposed to light and naturally present AA [12]. However, the decomposition of anthocyanins in mulberry is accelerated by constant light (5000 lx, 50 Hz) or the presence of AA [13]. Therefore, this study hypothesized that the three amino acids L-aspartic acid, L-valine, and L-proline could enhance the stability of mulberry anthocyanins under constant light (5000 lx, 50 Hz) or AA. The intermolecular interactions between L-proline and C3G were further investigated using FT-IR, ^1^H NMR, XRD, and molecular docking analyses, as well as molecular dynamics modes. The DPPH and ABTS radical scavenging capacity of L-Proline/C3G during in vitro digestion was also investigated. The results of this study contribute to explaining the mechanism of the interaction between C3G and L-proline at the molecular level and improving the stability and bioactivity of anthocyanins in mulberry fresh fruits and beverages.

## 2. Results and Discussion

### 2.1. Effects of Amino Acids on Mulberry Anthocyanins in the Presence of Constant Light and AA

**Figure 1:**
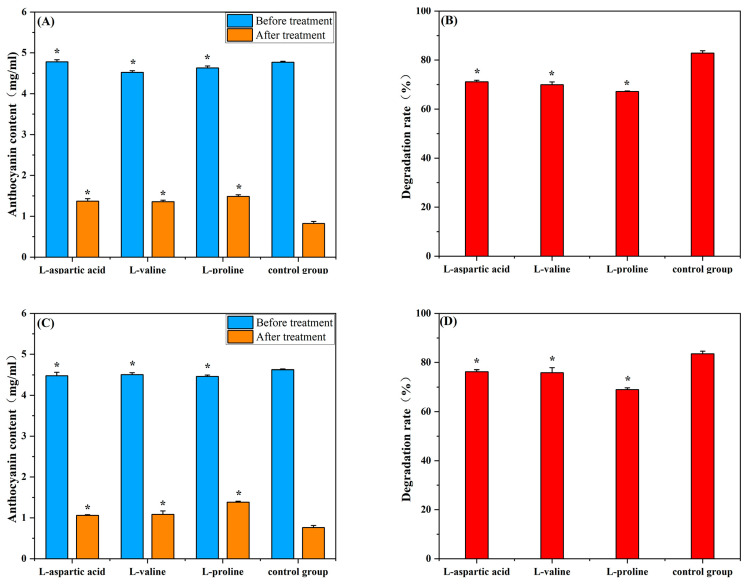
Effect of amino acids on anthocyanin content and degradation rate of mulberry after 7 days of constant light (5000 Lux, 50 Hz) and ascorbic acid irradiation. The control group was mulberry anthocyanins without amino acids. (**A**) Effect of amino acids on anthocyanin content of mulberry after 7 days of exposure to constant light (5000 Lx, 50 Hz); (**B**) Effect of amino acids on the degradation rate of anthocyanins in mulberry after 7 days of exposure to constant light (5000 Lx, 50 Hz); (**C**) Effect of amino acids on anthocyanin content of mulberry after 7 days of AA addition; (**D**) Effect of amino acids on the degradation rate of anthocyanins in mulberry after 7 days of AA addition. * *p* < 0.05 vs. control group (no added amino acids).

Multiple studies have shown that amino acids have been used as co-pigmenting substances to slow down the degradation of anthocyanins in the processing of mulberry products [14]. As shown in Figure 1A,B, the degradation rate of C3G in the anthocyanin group was significantly higher than that of C3G in the three amino acid groups under constant light (5000 lx, 50 Hz) for 7 d (*p* < 0.05). The degradation rate of C3G in the control group was 82.83 ± 1.0%, while the degradation rate of C3G in the L-aspartic acid, L-valine, and L-proline groups decreased to 71.14 ± 0.62%, 67.97 ± 1.11% and 67.19 ± 0.21%, respectively. This result showed that the L-aspartic acid, L-valine, and L-proline groups had significant protective effects (*p* < 0.05) on the degradation of C3G. The L-proline group showed the strongest protective effect against C3G degradation under constant light (5000 lx, 50 Hz).

The degradation rate of C3G in the control group was higher than that of C3G in the amino acid group in the presence of AA (Figure 1C,D) (*p* < 0.05). The degradation rate of C3G was 83.5 ± 1.07% in the control group. However, the degradation rate of C3G in the L-asparagine, L-valine, and L-proline groups decreased to 76.27 ± 0.77%, 75.9 ± 1.21%, and 68.97 ± 0.74%, respectively. These results indicated that L-aspartic acid, L-valine, and L-proline significantly inhibited the degradation of mulberry anthocyanin under the effect of AA or constant light. L-Proline showed the strongest protective effect against C3G degradation. The study found that aspartic acid, methionine, and tryptophan enhanced the stability of rhododendron anthocyanins through intermolecular interactions [15]. Zhang [16] found that L-proline effectively improved the color stability of C3G. This was in agreement with the findings of the current research, which indicated that L-proline was the most efficient co-pigment for increasing the anthocyanin stability in mulberry.

### 2.2. Effects of Amino Acids on Degradation Kinetics of Mulberry Anthocyanins

The degradation kinetics and half-life of mulberry anthocyanins under constant light (5000 lx, 50 Hz) or AA are shown in Table 1. Compared with the control group, the L-aspartic acid, L-valine, and L-proline groups had lower degradation rate constants and longer half-lives when exposed to constant light (5000 lx, 50 Hz) or AA. Among these groups, the L-proline group exhibited the lowest degradation rate constants for anthocyanins (*k* = 0.19313 and 0.17852) and the longest half-lives (*t*_1/2_ = 3.59 d and 3.88 d) under constant light (5000 lx, 50 Hz) or AA, respectively, indicating that L-proline provided the most effective protection against the degradation of mulberry anthocyanins. The protective effect of L-proline on mulberry anthocyanins might be due to the molecular interactions between C3G and L-proline through hydrophobic and hydrophilic interactions, including van der Waals forces and hydrogen bonding, which enhanced the stability of anthocyanins [17,18]. Thus, the molecular interaction between C3G and L-proline was further investigated through FT-IR, ^1^H NMR, XRD, and molecular docking analyses, as well as molecular dynamics modes.

### 2.3. Fourier Infrared Spectroscopy

**Figure 2:**
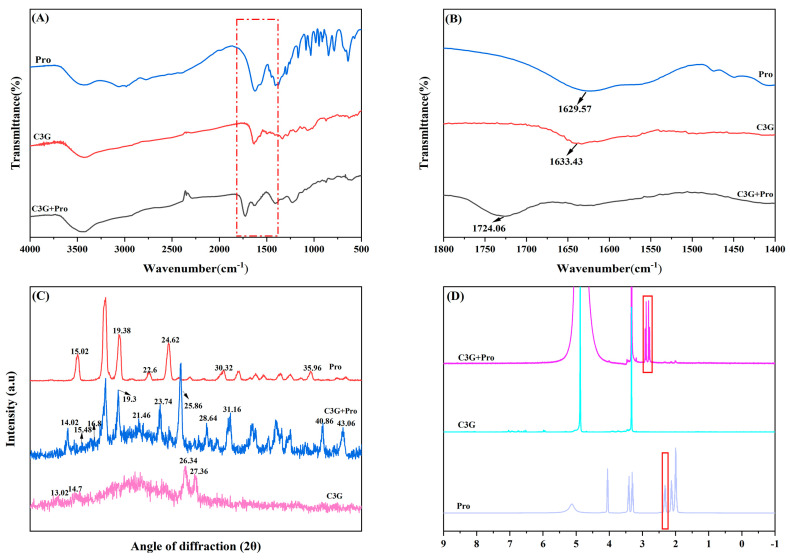
FT-IR spectra, XRD patterns and ^1^H NMR spectra of L-proline, C3G and L-proline/C3G mixture. (**A**) FT-IR spectra at the wavelength of 4000 cm^−1^–400 cm^−1^; (**B**) the enlarged scale of FT-IR spectra at the wavelength of 1800 cm^−1^–1400 cm^−1^; (**C**) XRD patterns; (**D**) ^1^H NMR spectra.

**Figure 3:**
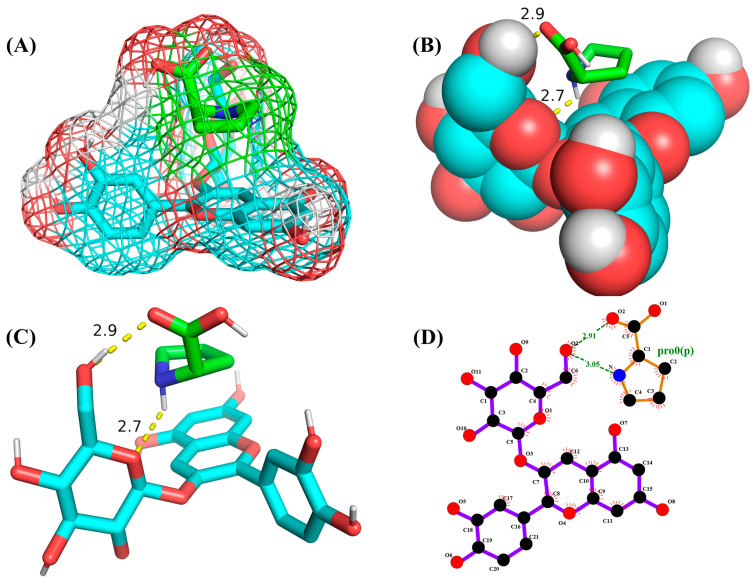
Optimal conformations of C3G and L-proline. The color coding of the interactions is as follows: conventional hydrogen bonding (yellow dashed line). (**A**) Three-dimensional molecular structure of L-proline and C3G in a wireframe grid; (**B**) Diagram of the molecular structures of L-proline and C3G as displayed in the space-filling model; (**C**) Molecular structure diagram of L-proline-C3G; (**D**) Two-Dimensional Molecular Structure of L-Proline-C3G.

The interaction between L-proline and C3G molecules was analyzed by the characteristic peaks of the FT-IR spectra (Figure 2A,B). FT-IR spectra of L-proline, C3G, and L-proline/C3G mixtures in the 4000–400 cm^−1^ wavenumber range are shown in Figure 3A,B. The infrared spectral region of the C=O bond was between 1600 and 1900 cm^−1^. The characteristic peak of C3G was at 1633.43 cm^−1^, and the peak of L-proline was at 1629.57 cm^−1^. The peak of C3G/L-proline mixtures was at 1724.06 cm^−1^, indicating that the peak of C3G was shifted after the addition of L-proline. The study found that a strong interaction between C3G and L-proline was confirmed by the appearance of new characteristic absorption peaks through infrared spectrograms [16]. The red shift of the characteristic peaks of the L-tryptophan-anthocyanin complex indicated the existence of hydrogen bonding and hydrophobic interactions between L-tryptophan and anthocyanins [8]. The shift of the characteristic peaks of C3G suggested the interaction between C3G and L-proline, which was related to the presence of hydrogen bonding and hydrophobic interactions.

### 2.4. XRD Pattern

The X-ray diffraction patterns of L-proline, C3G, and L-proline/C3G mixtures were determined when L-proline was mixed with C3G (Figure 2C). XRD analysis indicated that L-proline exhibited distinct peaks at 15.02, 19.38, 22.6, 24.62, 30.32, and 35.96. C3G showed distinct peaks at 13.02, 14.7, 26.34, and 27.36. When L-proline was mixed with C3G, new peaks appeared at 14.02, 16.8, 25.86, 28.64, 31.06, 40.86, and 43.06. According to the changes in the peak position of L-proline, we determined that the interaction between L-proline and C3G resulted in the formation of a new crystal structure. The decrease in the kurtosis of L-proline in the XRD pattern of the L-proline/C3G mixtures indicated a decrease in the degree of ordering of the crystal structure, suggesting that the addition of L-proline had an impact on the crystal structure of C3G.

### 2.5. NMR Hydrogen Spectrum Analysis

The NMR hydrogen spectral mapping showed the location of the hydrogen signals in the material, which contributed to an inference of the associated structural changes. Figure 2D shows the NMR hydrogen spectral profiles of C3G, L-proline, and L-proline/C3G mixtures. We observed a broad peak in the range of 2.27–2.39 ppm, which represented the unique -NH- group of L-proline. However, the hydrogen signal of L-proline after the addition of C3G generated a new absorption peak in the region of 2.75–2.94 ppm (Figure 2D), which implied that the hydrogen bond or other intermolecular forces were formed between L-proline and C3G. Nie et al. [10] found by NMR hydrogen spectroscopy that the interaction of anthocyanins and L-methionine was due to the formation of a hydrogen bond between L-methionine and C3G, which was consistent with the hydrogen NMR mapping results of the L-proline/C3G mixtures. The above results suggested that there was an interaction between L-proline and C3G, which might be associated with the formation of hydrogen bonds and van der Waals forces.

### 2.6. Molecular Docking Analysis

Molecular docking is used to predict molecular conformations and interactions [19]. Molecular docking can be used to calculate the interaction energy between molecules, with a higher negative binding energy indicating a tighter and more stable ligand–receptor interaction [20]. To further confirm the interaction between C3G and L-proline, all spatial conformations were visualized. It was found that L-proline could form a suitable conformation with C3G (Table 2). The conformational energies of interaction between C3G and L-proline as obtained by molecular docking ranged from −1.5 to −1.7 kcal/mol, indicating the strong interaction between C3G and L-proline that was formed by hydrogen bonding between the H atoms of the hydroxyl group on C3G and the O atoms of the carbon–oxygen double bond in L-proline. Interaction energy analysis revealed a strong interaction between L-proline and C3G with an interaction energy of −1.7 kcal/mol. The most common π-π stacking interactions are those between benzene rings, which have energy magnitudes of about 1 to 50 kJ/mol^−1^, mostly around and below 10 kJ/mol^−1^. The lowest affinity and root–mean–square deviation (RMSD) values of the two models were compared to determine the optimal conformation. Despite the RMSD value of 0 for model 1, model 2 was considered to be the optimal conformation because of the absence of hydrogen bonding [21].

### 2.7. Molecular Dynamics Simulation Results

As shown in Figure 3, L-proline effectively bound to the active pocket of C3G to form a three-dimensional cavity. Three-dimensional interaction analysis revealed that the carboxyl group of L-proline was able to form more hydrogen bonding interactions with C3G, with hydrogen bonding distances of 2.9 and 2.7 Å. Thus, hydrogen bonding was the main intermolecular interaction that provided stability to the bound conformation of C3G and L-proline. Hydrogen bonding interactions between anthocyanins and amino acid molecules have been shown to enhance anthocyanin stability [22], which is consistent with our results.

The MM2 force field is an empirical force field based on experimental data and quantitative chemical theory that is used to characterize interactions between atoms and conformational changes within molecules. Chemical changes driven by thermodynamics represent the energy relationship between different chemical changes [10,23]. The MM2 formula is used to obtain the molecular dynamics by using molecular dynamics modeling [24]. The potential energy analysis of the L-proline/C3G mixtures showed that the stretching, bending, and torsion energies of the intramolecular interactions of L-proline and C3G decreased at the end of the molecular dynamics simulations compared to the beginning of the simulations (Table 3). The bending energy of L-proline/C3G mixtures was significantly decreased according to the potential energy analysis; this might be a result of the instability of the aromatic ring in C3G, where the б bonds could be easily twisted to produce an optimal energy distribution [25]. As shown in Table 3, the van der Waals interaction energy of more than three atoms between the L-proline and C3G molecules, which constituted the main portion of the stabilization energy of the L-proline and C3G molecular clusters, was significantly reduced. Nie et al. [10] found that the stretching energy, bending energy and torsion energy were decreased at the end of the kinetic simulation and the Van der Waals interaction energy of more than three atoms had been significantly reduced. This is in agreement with our results. This result indicated that van der Waals forces improved the C3G stability through the interaction between L-proline and C3G. In this study, the interaction between C3G and L-proline formed by hydrogen bonding and van der Waals forces improved the stability of C3G, which might be due to enhanced stabilization of the cationic chromophores of the anthocyanins, preventing the anthocyanins from interfering with water molecules and slowing color fading throughout the storage process [26,27,28].

### 2.8. Analysis of the Results of In Vitro Assay for Digestive Antioxidant Activity

**Figure 4:**
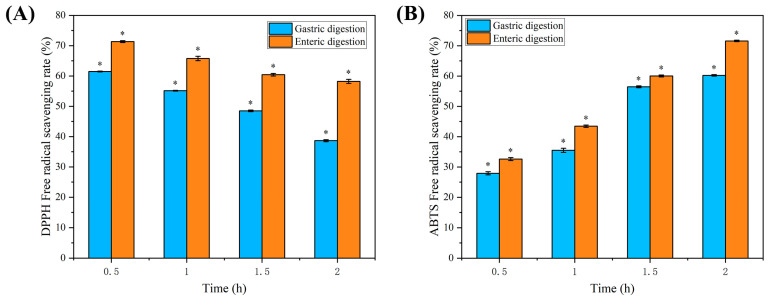
Effect of simulated digestion on the antioxidant capacity of C3G/Pro groups. (**A**) Effect of simulated digestion on 2,2-Diphenyl-1-picrylhydrazyl (DPPH) free radical scavenging capacity of C3G/Pro group. (**B**) Effect of simulated digestion on 2,2′-Azino-bis(3-ethylbenzothiazoline-6-sulfonic acid) (ABTS) free radical scavenging capacity of C3G/Pro group. * *p* < 0.05 vs. Comparison of different times of gastric or intestinal digestion.

Figure 4A indicated that the 2,2-Diphenyl-1-picrylhydrazyl (DPPH) radical scavenging rate of the C3G/Pro group gradually decreased with prolonged gastric digestion time. The DPPH radical scavenging rate of the C3G/Pro group was decreased by 23.22% from 0.5 h to 2 h of gastric digestion. During the intestinal digestion process, the DPPH radical scavenging rates of the C3G/Pro group were significantly higher than that observed in the gastric digestion phase. The enzymes present in the intestinal fluids aided in liberating phenolic substances from their anthocyanin-bound form, which in turn boosted the scavenging of DPPH free radicals [29,30,31]. Therefore, the C3G/Pro group showed increased scavenging of DPPH radicals.

Figure 4B indicated that the 2,2′-Azino-bis(3-ethylbenzothiazoline-6-sulfonic acid) (ABTS) radical scavenging capacity of the C3G/Pro group increased by 32.30% from 0.5 h to 2 h of gastric digestion. The ABTS radical scavenging capacity of the C3G/Pro group significantly increased by 41.84% from 0.5 h to 2 h of intestinal digestion. The ABTS radical scavenging capacities of the C3G/Pro group in the intestinal digestion phase were significantly higher than that in the gastric digestion phase, which may be attributed to the deprotonation of phenolic hydroxyl groups in the aromatic rings of C3G under weakly alkaline pH conditions [32], which resulted in an increased scavenging capacity of the C3G/Pro group for ABTS radicals.

## 3. Materials and Methods

### 3.1. Materials and Ingredients

L-Aspartic acid, L-valine, L-proline, and AA, were purchased from Shanghai McLean Biochemical Technology Co., Ltd. (Shanghai, China). Cyanidin-3-*O*-glycoside standard (purity ≥ 95%) was purchased from ShanghaiYuanye Biotechnology Co., Ltd. (Shanghai, China).

### 3.2. Mulberry Anthocyanin Extraction

The anthocyanins of mulberry were extracted by microwave-assisted two-phase aqueous extraction following the method of Wang et al. [33]. The phase diagram of the aqueous ethanol–ammonium sulfate system was determined using the titration method [34]. First, we added 20 mL of 250 mg/mL ammonium sulfate solution to a 50 mL beaker and titrated it with ethanol while stirring until cloudy. Distilled water was then added to clarify the solution. One gram of mulberry powder and 45 mL of extractant were added to a three-well flask and stirred well. Microwave-assisted extraction of solutions was performed using the microwave chemical reactor at atmospheric pressure. After extraction, the samples were rapidly cooled to 25 °C by placement in a pool of ice and water. The samples were then separated using a liquid-separating funnel.

Purification of mulberry anthocyanin extract was performed using NKA-9 macroporous resin with reference to Yuan et al. [35]. We added 25 g of activated NKA-9 macroporous resin to the chromatographic column (30 × 30 mm) [36]. The anthocyanin solution pH was controlled to 3.5, and the samples were sorbed at a flow rate of 0.001 L/min. The resin was then washed with distilled water until it was colorless, and eluted with 600 mL/L of ethanol. The samples were subjected to rotary evaporation at 45 °C to remove ethanol. The resulting solution was then freeze-dried to produce the mulberry anthocyanin extract powder.

### 3.3. Preparation of Mulberry Anthocyanin–Amino Acid Solution

An aqueous solution with amino acids (L-aspartic acid, L-valine, and L-proline) was produced by diluting the composition in powder form in distilled water and stirring until complete dissolution. The solution was adjusted to pH 3.0 using citrate buffer. The anthocyanic extract was added to each of the three amino acid solutions and stirred for 30 min to obtain a mixed solution of 2.0 mg/mL amino acids (aspartate, valine, and proline) and 0.5 mg/mL anthocyanic extract. The three prepared mulberry anthocyanin-amino acid solutions were stored under light at about 25 °C for 7 days to observe the stability of amino acids against degradation of mulberry anthocyanin solutions.

### 3.4. Anthocyanin—Amino Acid—AA Mixed Solution Preparation

The model beverage system from Wang et al. [37] was used and modified slightly. AA was mixed with the prepared mixture of anthocyanins and amino acids, and the mixture was stirred until complete dissolution. The final mixed solution contained a 4:1:2 ratio of amino acid, mulberry anthocyanin, and AA. We readjusted the solution at pH 3.0 as needed. All the prepared mixture solutions were stored at 25 °C and protected from light for 7 days to measure the effect of AA on the stabilization of the three mulberry anthocyanin–amino acid solutions.

### 3.5. Estimation of Mulberry Anthocyanins

The pH difference technique was used to measure the anthocyanin content [38]. Anthocyanin-amino acid solutions diluted with potassium chloride buffer (pH 1.0) and sodium acetate buffer (pH 4.5) were prepared in the presence of constant light (5000 lx, 50 Hz) and AA. The diluted mixed solution was incubated in the dark for a period of 15 min. The absorbance of the solution was then measured at 520 nm and 700 nm using a UV-Vis spectrophotometer (UV-3600, Shimadzu Corporation, Tokyo, Japan).

The anthocyanin concentration was then estimated using Equations (1) and (2):

*A* = (*A*_1_ − *A*_2_) pH1.0 − (*A*_1_ − *A*_2_) pH4.5 (1)

AC = *A* × MW × DF × 10^3^/(ε × l) (2)

where *A* represents the absorbance, *A*_1_ represents the absorbance at 520 nm, and *A*_2_ represents the absorbance at 700 nm. MW is the molecular weight of cyanidin-3-*O*-glucoside (449.2 g/mol), DF is the dilution factor, ε is the molar extinction coefficient of cyanidin-3-*O*-glucoside (26,900), and l is the length of the reaction beaker (1 cm).

Equation (3) was used to calculate the degradation rate of anthocyanins:

*D* (%) = (*B*_0_ − *B*_1_)/*B*_0_ × 100 (3)

where *D* is the rate of anthocyanin degradation (%), *B*_0_ is initial anthocyanin content, and *B*_1_ is the anthocyanin content after storage.

### 3.6. Analysis of Mulberry Anthocyanin Degradation Kinetics

A previously reported first-order model was used to evaluate the degradation kinetics of mulberry anthocyanins in solution under constant light conditions or in the presence of AA [18]. The values of the anthocyanin half-time in mixed solutions were determined according to the methodology of Pham et al. [39].

The primary reaction is modeled as shown in Equation (4):

ln (*N_t_*/*N*_0_) = −*k* × t (4)

The first-order response model *t*_1/2_ value is calculated using Equation (5):

*t*_1/2_ = −ln0.5 × *k*^−1^ (5)

In the above equation, *t* is the reaction time, *N_t_* is the absorbance at reaction time *t*, *N*_0_ is the absorbance at initial time, and *k* is the first kinetic constant. Half-time (*t*_1/2_) is the time it takes for anthocyanins to decay to half their original amount.

### 3.7. Spectrum Instrumental Analysis

#### 3.7.1. FT-IR Analysis

The same procedure for the amino acid/mulberry anthocyanin mixtures was used to prepare the L-proline/C3G mixture and its lyophilized samples. A dry-powder sample (L-proline, C3G, or L-proline + C3G) weighing 1 mg was combined with 200 mg of KBr and compacted into translucent discs [40]. After that, Fourier infrared spectra were acquired in the 4000–400 cm^−1^ range using an FT-IR spectrometer with a resolution of 4 cm^−1^. The spectral data were acquired with OPUS software (Vertex 70, Bruker Instrument Co., Ltd., Berlin, Germany). Spectral data of each sample were analyzed by Origin 2019B software (OriginLab Corporation, Northampton, MA, USA) after three scans.

#### 3.7.2. X-ray Diffraction Analysis

We performed XRD analysis on the dried L-proline, C3G, and L-proline/C3G mixtures. We recorded their XRD patterns using a general-purpose XD-2 automatic X-ray powder diffractometer with Cu Kα radiation (λ = 0.5141 nm). The value of θ was varied from 10° to 80° in increments of 0.0167°. The voltage used for operation was 30 kV, while the current used was 40 mA.

#### 3.7.3. ^1^H NMR Analysis

We used deuterated methanol as a solvent to dissolve 5 mg of the dried L-proline, C3G, and L-proline/C3G mixtures [41]. The ^1^H NMR spectra were obtained with an Anance III (500 M) spectrometer operated at 500 MHz.

### 3.8. Molecular Docking

The docking procedure was similar to that of Xing et al. [21] with minor modifications. Ligand–receptor docking was performed using the molecular structures of C3G (PubChem CID: 441667) and L-proline (PubChem CID: 145742) from the PubChem Molecular Database. C3G and L-proline were processed using AutoDockTools 1.5.6 software. Water was deleted, hydrogen was added, nonpolar hydrogen atoms were merged, and the data were saved in pdbqt format. The combination of C3G and L-proline was blindly docked using AutoDock Vina 4.2 software to produce the best binding conformation. Results were obtained with AutoDockTools 1.5.6 and visualized with PyMol. In molecular docking, the optimal result usually refers to the formation of a stable binding conformation between the ligand and the receptor that has a low free energy of binding. The optimal outcomes achieved by the aforementioned methodologies were essential for calculating and analyzing molecular dynamics [20].

### 3.9. Molecular Dynamics Approach to Simulation Calculations

Molecular dynamics calculations (MM2) were performed with reference to Nie et al. [10]. Chem3D 20.0 software (Cambridge, UK) generated three-dimensional molecular models. Force field approximations were employed to minimize energy and to conduct molecular dynamics simulations. The equations of state and characteristic parameters of various atoms and chemical bonds were determined using the advanced Alliger MM2 force field.

A set of classical molecular mechanics potential energy equations was used to characterize the energy of a molecule when subjected to the MM2 force field. The assumption was that the molecule would be in a state of isolation in a vacuum. Equation (6) was used to calculate the total energy of the molecule:

*F* = *F*_s_ + *F*_b_ + *F*_t_ + *F*_v_ + *F*_e_ (6)

where *F*_s_, *F*_b_, and *F*_t_ are the bond interactions, *F*_s_ is the stretch energy between bonded atoms, *F*_b_ is the bending energy between bonded pairs, *F*_t_ is the torsional energy of neighboring atoms, *F*_v_ is the interatomic van der Waals interaction energy, and *F*_e_ is the electrostatic interaction energy. The last two terms of the equation represent non-bonding interactions. Because of these interactions, a stretch–bend term was included to account for the increased bond length due to the increased bond angle.

The movements observed molecular in the MM2 force field were investigated using Newton’s equations of motion. Molecular dynamics is used primarily for studying how model molecules change conformation and local vibrations, with the goal of finding the most energetically favorable state among all possible states of the molecule. The analog temperature was set to 300 K. The simulation lasted 20 fs, and we controlled the system temperature within the range of 300 ± 15 K.

### 3.10. Preparation of Simulated Digested Samples

The method of Li et al. [30] was slightly modified. In a 100-mL volumetric flask, a solution of 0.4 g of pepsin in 90 mL of a 9 mg/mL NaCl solution should be prepared. The pH was adjusted to 2 using 1 M HCl, and the volume was brought to 100 mL. Subsequently, the mixture was subjected to incubation in a shaker at 250 rpm and 37 °C for a period of 2 h. At 0.5, 1.0, 1.5, and 2.0 h, samples were obtained for analysis. The antioxidant activity of the digest was assessed by determining the ABTS and DPPH radical scavenging rates.

After this, the gastric digestive reaction was neutralized to pH 6.5 with NaOH to halt the gastric digestion. Subsequently, 20 mL of intestinal digestive solution consisting of 225 mg of trypsin, 225 mg of bile extract, and 90 mL of a 9 mg/mL NaCl solution was added. The pH was adjusted to 7 using 1 M NaOH, and the volume was fixed at 100 mL. The sample solution continued to react for 2 h, and the antioxidant activities of the digested solutions were determined by sampling at 0.5, 1.0, 1.5, and 2.0 h into the reaction.

### 3.11. Determination of Antioxidant Activity

#### 3.11.1. Assay for DPPH Free Radical Scavenging Capacity

In this study, we employed a method originally proposed by Ma et al. [42], with minor modifications. A 0.5 mL portion of the solution to be measured was taken at 0, 0.5, and 1.0 h of the simulated gastric and intestinal digestion phases. A total of 2 mL of a 0.06 mmol/L DPPH ethanol solution was added, mixed homogeneously, and then reacted for 0.5 h in the darkness. The sample solution’s absorbance was recorded at 517 nm (A1), while the absorbance A0 was measured after 0.5 mL of deionized water was combined with 2 mL of the DPPH ethanol solution. The absorbance A0 was determined for a mixture of 0.5 mL deionized water with 2 mL of DPPH ethanol solution, and the absorbance A2 was measured for a combination of 0.5 mL of the sample solution with 2 mL of anhydrous alcohol. The DPPH radical scavenging rate was calculated in accordance with the following formula:

$$DPPH\;RSA\left(\%\right)=[1-\left(A_{1}-\frac{A_{2}}{A_{0}}\right)]\times 100$$

where *A*_0_ represents the absorbance value of the blank control, *A*_1_ is the absorbance value of the sample, and *A*_2_ is the absorbance value of the reference solution.

#### 3.11.2. Assay for ABTS Free Radical Scavenging Capacity

In this research, we adopted a technique initially suggested by Ma et al. [42], incorporating slight alterations. In order to create the ABTS reserve solution, 7.0 mmol/L ABTS aqueous solution was mixed with 2.45 mmol/L potassium persulfate at equal volumes (1:1). This mixture was then reacted for 16 h in the absence of light, after which it was diluted with anhydrous ethanol in the absence of light to reach an absorbance of 0.70 at a wavelength of 734 nm. This solution was then used to create the ABTS reaction solution. A total of 200 μL of the solution to be measured was to be taken from the simulated gastric digestion and intestinal digestion stage. This was to be combined with 3 mL of ABTS reaction solution and reacted for 1 h in the dark at room temperature. The same conditions were to be used for the measurement of the absorbance of 200 μL of anhydrous ethanol and 3 mL of ABTS reaction solution, *A*_0_. The subsequent equation should be applied to determine the ABTS radical scavenging rate (%):

$$ABTS\;RSA\left(\%\right)=\frac{\left(A_{0}-A_{1}\right)}{A_{0}}\times 100$$

where *A*_0_ represents the absorbance value of the blank control, while *A*_1_ denotes the absorbance value of the sample.

### 3.12. Data Handling and Statistical Analysis

The assay was replicated three times, and the results are reported as the mean ± standard deviation of three independent measurements. The software SPSS Statistics 27, developed by the company IBM, USA, was used for the statistical study of all the experimental data. Differences between treatment averages were assessed by the ANOVA Tukey test (*p* < 0.05).

## 4. Conclusions

This study investigated the effect of L-aspartic acid, L-valine, and L-proline on the stability of mulberry anthocyanins under constant light (5000 lx, 50 Hz) or AA conditions. L-Aspartic acid, L-valine, and L-proline significantly improved the stability of mulberry anthocyanin under the effect of AA or constant light, especially L-proline. The protective effect of L-proline on the stability of cyanidin-3-*O*-glycoside could be attributed to the interaction between C3G and L-proline formed through hydrogen bonding and van der Waals forces, according to the results of FT-IR, XRD, ^1^H NMR, and molecular docking analyses, as well as molecular dynamics mode (MM2). The results of the in vitro digestion assay showed different antioxidant properties of the C3G/Pro group in the in vitro digestion assay. This study emphasized that L-proline improves the color stability of mulberry anthocyanin-rich beverages and that hydrogen bonding is the main intermolecular interaction that improves the stability of C3G and L-proline. Moreover, the addition of L-proline helped to improve the bioavailability of C3G. This study provides an important theoretical and experimental basis for the application of mulberry anthocyanins and a new strategy for the food industry to improve the stability and nutritional value of mulberry anthocyanins.

## Figures and Tables

**Table 1:** Degradation kinetic (*k*) and half time (*t*_1/2_) of mulberry anthocyanins affected by amino acid addition under constant light (5000 lx, 50 Hz) or ascorbic acid presence.

	Constant Light			Ascorbic Acid		
	*k* (day^−1^)	*t*_1/2_ (day)	*R* ^2^	*k* (day^−1^)	*t*_1/2_ (day)	*R* ^2^
Control group	0.23133	2.9964	0.95705	0.23866	2.9043	0.96410
L-aspartic acid group	0.19740	3.5114	0.98726	0.20741	3.3419	0.86409
L-valine group	0.19885	3.4858	0.90633	0.21639	3.2032	0.94333
L-proline group	0.19313	3.5890	0.94759	0.17852	3.8827	0.92333

Control group: mulberry anthocyanins without amino acid addition; L-aspartic acid group: mulberry anthocyanins with L-aspartic acid addition; L-valine group: mulberry anthocyanins with L-valine addition; L-proline group: mulberry anthocyanins with L-proline addition. *R*^2^: Correlation coefficient.

**Table 2:** Molecular docking models between cyanidin-3-*O*-glucoside and L-proline.

MODE	Affinity (kcal/mol)	Distance from Best Mode		Binding Conformation
		Rmsd l.b.	Rmsd u.b.	
1	−1.7	0	0	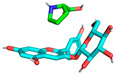
2	−1.7	7.116	9.627	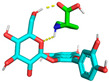
3	−1.6	5.650	8.191	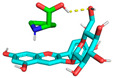
4	−1.6	5.437	7.887	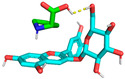
5	−1.6	3.321	8.037	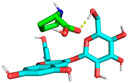
6	−1.6	6.442	9.144	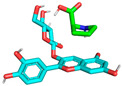
7	−1.6	6.994	9.600	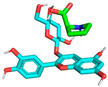
8	−1.6	5.841	9.045	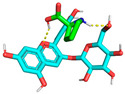
9	−1.5	7.258	8.747	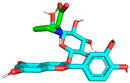

**Table 3:** Potential energy of anthocyanin-amino acid model at the initial and termination of molecular dynamic simulations.

	Stretching	Bending	Stretch-Bending	Torsion	Non-1,4 VDW	1,4 VDW	Dipole/Dipole	Total
Initial	0.5107	9.2850	0.3184	17.2005	−21.5507	16.1389	−15.6499	16.2466
Termination	0.3996	5.8711	0.2272	15.8223	−24.0872	13.5361	−11.6041	0.1649

Non-1,4 VDW represents the energy for the through-space Van der Waals interaction between pairs of atoms that are separated by more than three atoms; 1,4 VDW means Van der Waals interaction energy.

## Data Availability

The raw data supporting the conclusions of this article will be made available by the authors upon request.
